# Peroxisome proliferator-activated receptor-gamma: potential molecular therapeutic target for HIV-1-associated brain inflammation

**DOI:** 10.1186/s12974-017-0957-8

**Published:** 2017-09-08

**Authors:** Amila Omeragic, Md Tozammel Hoque, U-yeong Choi, Reina Bendayan

**Affiliations:** Department of Pharmaceutical Sciences, Leslie Dan Faculty of Pharmacy, University of Toronto, 144 College Street, Toronto, ON M5S 3M2 Canada

**Keywords:** HIV-1_ADA_ gp120, PPARγ, HIV-1, Brain inflammation, Cytokines, Glutamate

## Abstract

**Background:**

Despite the use of combination antiretroviral therapy for the treatment of HIV-1 infection, cognitive impairments remain prevalent due to persistent viral replication and associated brain inflammation. Primary cellular targets of HIV-1 in the brain are macrophages, microglia, and to a certain extent astrocytes which in response to infection release inflammatory markers, viral proteins [i.e., glycoprotein 120 (gp120)] and exhibit impaired glutamate uptake. Peroxisome proliferator-activated receptors (PPARs) are members of the nuclear receptor superfamily of ligand-activated transcription factors. Compelling evidence suggests that PPARγ exerts anti-inflammatory properties in neurological disorders. The goal of this study was to examine the role of PPARγ in the context of HIV-1_ADA_ gp120-induced inflammation in vitro, in primary cultures of rat astrocytes and microglia, and in vivo, in a rodent model of HIV-1_ADA_ gp120-associated brain inflammation.

**Methods:**

Primary mixed cultures of rat astrocytes and microglia were treated with PPARγ agonists (rosiglitazone or pioglitazone) and exposed to HIV-1_ADA_ gp120. Inflammatory cytokines and indicator of oxidative stress response (TNFα, IL-1β, iNOS) were measured using qPCR, and glutamate transporter (GLT-1) was quantified by immunoblotting. In vivo, rats were administered an intracerebroventricular injection of HIV-1_ADA_ gp120 and an intraperitoneal injection of PPARγ agonist (rosiglitazone) or co-administration with PPARγ antagonist (GW9662). qPCR and immunoblotting analyses were applied to measure inflammatory markers, GLT-1 and PPARγ.

**Results:**

In primary mixed cultures of rat astrocytes and microglia, HIV-1_ADA_ gp120 exposure resulted in a significant elevation of inflammatory markers and a decrease in GLT-1 expression which were significantly attenuated with rosiglitazone or pioglitazone treatment. Similarly, in vivo, treatment with rosiglitazone reversed the gp120-mediated inflammatory response and downregulation of GLT-1. Furthermore, we demonstrated that the anti-inflammatory effects of PPARγ agonist rosiglitazone were mediated through inhibition of NF-κB.

**Conclusion:**

Our data demonstrate that gp120 can induce an inflammatory response and decrease expression of GLT-1 in the brain in vitro and in vivo. We have also successfully shown that these effects can be reversed by treatment with PPARγ agonists, rosiglitazone or pioglitazone. Together our data suggest that targeting PPARγ signaling may provide an option for preventing/treating HIV-associated brain inflammation.

**Electronic supplementary material:**

The online version of this article (10.1186/s12974-017-0957-8) contains supplementary material, which is available to authorized users.

## Background

The entry of the human immunodeficiency virus (HIV-1) into the central nervous system (CNS) occurs early in the course of infection either as a cell-free virion or encased within infected macrophages [1]. Some reports also document that HIV-1 can cross the blood-brain barrier (BBB) through a receptor-mediated transcytosis possibly using the mannose-6-receptor [2]. In the CNS, the major targets of HIV-1 are mononuclear phagocytes (e.g., perivascular macrophages and brain resident microglial cells) and to lesser degree astrocytes. In response to HIV-1, microglia and astrocytes become activated and secrete pro-inflammatory cytokines [i.e., tumor necrosis factor-α (TNFα), interleukin-1β (IL-1β), interleukin-6 (IL-6), interleukin-8 (IL-8)] and neurotoxins [i.e., arachidonic/quinolinic acid and metabolites, platelet-activating factor, neurotoxic amines, reactive oxygen species (ROS), nitric oxide (NO), and glutamate] [3]. Although neurons do not appear to be directly infected by HIV-1, the prolonged exposure to inflammatory, neurotoxic, and oxidative stress markers during infection can cause neuronal injury and death [4]. HIV-1 viral proteins such as envelope glycoprotein (gp120), transactivator of transcription (Tat), and viral protein R (Vpr) are also known to be neurotoxic upon release from infected cells [3, 5]. It has been postulated that mechanisms triggering neuronal apoptosis involve viral protein interactions with neuronal chemokine receptors, excitotoxicity due to glutamate accumulation, caspase activation, loss of mitochondrial membrane potential, and DNA fragmentation [3]. We have previously demonstrated that R5 tropic HIV-1_ADA_ gp120 can mediate secretion of pro-inflammatory cytokines and oxidative stress markers by interacting with CCR5 chemokine receptor in primary cultures of human and rodent astrocytes, as well as in an in vivo rodent model of gp120-associated brain inflammation [6–8].

Despite receiving highly active antiretroviral therapy (HAART), up to 50% of infected individuals can develop HIV-1-associated neurocognitive disorders (HAND) which include memory, motor, and behavioral deficits, and can affect quality of life and mortality rate in these patients [9, 10]. The underlying mechanism for HAND remains poorly understood; however, a contributing factor may be chronic brain inflammation due to low level of HIV-1 replication in viral reservoirs such as microglia, and secretion or shedding of viral proteins (e.g., gp120, Vpr). Other contributing factors include age, low CD4^+^ T-cell nadir count, and comorbidities [9]. Currently, there are no effective treatments for HAND, and although HAART significantly prolongs lives of HIV-1-infected patients, variable effects have been reported on neurocognitive performance, and in some cases, certain antiretroviral drugs (ARVs) have been associated with neurotoxicity [11]. ARVs which are available for clinical use allow for systemic suppression of peripheral viral load; however, treating HIV in the brain remains a challenge partly due to the fact that several ARVs exhibit poor permeability across the BBB and into glial cells. In particular, protease inhibitors and nucleoside reverse transcriptase inhibitors display low brain penetration and do not reach therapeutic concentrations within the CNS, potentially allowing the brain to become a sanctuary for HIV-1 [12, 13]. Insufficient ARV concentrations in the brain could permit continuous HIV-1 replication and subsequent emergence of drug resistance viral strains despite acceptable control of the virus in the periphery [14]. In addition to low brain permeability, it is also important to note that the majority of ARVs do not exhibit direct anti-inflammatory properties. Therefore, identifying alternative therapeutic approaches that prevent release of neurotoxic factors from glial cells is critical for the treatment of HIV-associated brain inflammation and neurological disorders.

A variety of potential biomarkers have been identified in association with HAND. Several studies have shown, in HIV-infected individuals who develop HAND, markers of immune activation (neopterin, sCD14), cytokine expression (TNFα), and oxidative stress are more pronounced than in HIV individuals without cognitive impairments [15]. Abnormal glutamate homeostasis has also been observed in HIV-1-infected patients, where an increase in glutamate is observed in the cerebrospinal fluid (CSF) of patients with HAND as compared to healthy controls [16]. Furthermore, HIV-1 viral proteins Tat and gp120 have shown to decrease glial and synaptic uptake of glutamate [17–19].

With the increased prevalence of HAND among HIV-1-infected individuals, and the lack of effective therapy, it is critical to identify potential targets for the treatment of HAND [9]. In the past decade, there has been growing interest in the peroxisome proliferator-activated receptors (PPARs) ligand-activated transcription factors belonging to the nuclear receptors for steroid, thyroid hormones, and retinoids. These receptors play major roles in lipid homeostasis and glucose regulation [20]. Additionally, PPAR agonists can exhibit anti-inflammatory and antioxidant effects in several models of CNS disorders such as ischemic stroke and Alzheimer’s and Parkinson’s diseases [21–23]. Several studies have used both in vitro and in vivo models to demonstrate PPAR-mediated attenuation of the release of pro-inflammatory cytokines and oxidative stress markers [24]. In the context of HIV-1, there is also evidence suggesting that PPARγ and to a lesser extent PPARα agonists can play a neuroprotective role [5, 25, 26]. It has also been demonstrated that PPARγ agonist rosiglitazone can exhibit direct anti-HIV effects in different cell types such as Th1Th17 cells and monocyte-derived macrophages [27]; therefore, this isoform is of further interest. The protective anti-inflammatory effects of PPARγ have been shown to be partly mediated through transrepression of the redox regulated transcription factor nuclear factor kappa B (NF-κB) [28, 29].

The goal of this project was to investigate the role of PPARγ: (i) in suppression of inflammation and glutamate transporter 1 (GLT-1) dysregulation, in vitro, in primary cultures of rat microglia and astrocytes exposed to HIV-1_ADA_ gp120, and (ii) in vivo, in a rat model of HIV-1 associated brain inflammation.

## Methods

### Materials

HIV-1_ADA_ gp120 full-length recombinant protein (Clade B; R5-tropic) was obtained from immunodiagnostics Inc. (Woburn, Massachusetts, USA). PPARγ agonists rosiglitazone and pioglitazone and PPARγ antagonist 2-chloro-5-nitro-*N*-phenylbenzamide (GW9662) were purchased from Cayman Chemicals (Ann Arbor, Michigan, USA). Rabbit polyclonal anti-PPARγ (ab-6643), mouse monoclonal anti-CD11b/c (ab-1211), and rabbit polyclonal anti-EAAT2 (ab-41621) antibodies were purchased from Abcam Inc. (Boston, MA, USA). Rabbit polyclonal anti p-NF-κB p65^Ser536^ (sc-33020) and mouse monoclonal β–Actin (sc-47778) antibodies were obtained from Santa Cruz Biotechnology (Dallas, Texas, USA). Rabbit polyclonal antibody against glial fibrillary acidic protein (GFAP) and horse radish peroxidase (HRP) conjugated secondary antibodies (anti-mouse and anti-rabbit) were obtained from Sigma Aldrich (Missisauga, ON, Canada). Alexa fluor 488 and 594 (anti-rabbit or anti-mouse), 4′,6-diamidino-2-phenylindole hydrochloride (DAPI), western blot stripping solution, enhanced chemiluminescent reagents, and TRIzol were purchased from ThermoFisher Scientific (Waltham, MA, USA). High capacity reverse transcriptase cDNA synthesis kit and TaqMan FastMix were obtained from Applied Biosystems (Foster City, CA, USA) and Quanta Biosciences Inc. (Gaithersburg, Maryland, USA), respectively. Mixed astrocyte-microglia culture medium was prepared from minimum essential medium (OCI MEM H17 without antibiotic), gentamicin (Cat# 15750-060), Horse Serum (Cat# 16050-122) and Fetal Bovine Serum (Cat# 26140-079) from ThermoFisher Scientific (Waltham, MA, USA). 3-(4,5-dimethylthiazol-2-yl)-2,5-diphenyltetrazolium bromide (MTT) reagent was purchased from Sigma Aldrich (Mississauga, ON, Canada).

### Cell cultures

**Fig. 1:**
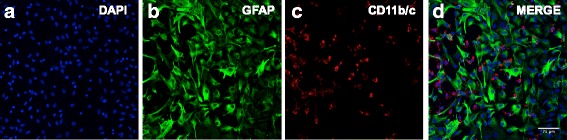
Immunocytochemical analysis of primary cultures of mixed rat astrocytes and microglia. Cells were immunostained with **a** DAPI, nuclear marker; **b** GFAP (1:200, dilution) astrocyte marker; (**c**) CD11b/c (1:20, dilution) microglial marker; **d** merged image. Cells were visualized using a confocal microscope (LSM 700, Carl Zeiss) operated with ZEN software using a 40× objective lens. Scale bar, 75 μM

Primary cultures of rat astrocytes and microglia were prepared as described previously in our laboratory [6] with a few modifications. All procedures were carried out in accordance with the University of Toronto Animal Care Committee and the Province of Ontario Animals for Research Act. In brief, whole brain isolates from 1- to 3-day-old neonatal Wistar rats (Charles River Laboratories, St. Constant, QC, Canada) were collected by cervical dislocation. Cerebral cortices were dissected and subjected to enzymatic digestion for 30 min in serum-free medium containing 2.0 mg/mL porcine pancreatic trypsin Sigma Aldrich (Mississauga, ON, Canada) and 0.005% DNase I purchased from Roche, Applied Science (Laval, QC, Canada). The tissue was then mechanically disaggregated using a cell dissociation kit from Sigma-Aldrich (Mississauga, ON, Canada) to yield a mixed glial cell suspension. The cell suspension was then centrifuged for 10 min at 100 g and resuspended in primary glial culture medium, which consisted of minimum essential medium supplemented with 5% horse serum, 5% fetal bovine serum, and 5 μg/ml gentamicin. The cells were plated onto 25-cm^2^ polystyrene tissue culture flasks (Sarstedt, St. Leonard, PQ, Canada) and incubated in fresh medium at 37 °C, in 5% CO2/95% air for 7 to 10 days until confluence was attained. For pure astrocyte cultures, the cells were then placed on an orbital shaker at 120 rpm for 6 h to remove microglia. Cells in culture were characterized for their purity and were assessed by morphological analysis and immunostaining for standard biochemical markers (e.g., glial fibrillary acidic protein for astrocytes, cd11b/c for microglia) (Fig. 1). K-562, the chronic myelogenous leukemia human cell lysate, was purchased from Santa Cruz Biotechnology (Dallas, Texas, USA) and was used as a positive control for p-NF-κB p65^Ser536^ in immunoblotting experiments. HepG2, hepatocyte carcinoma cell line, was purchased from ATCC (Manassas, Virginia, USA); cell lysates were prepared in our laboratory and used as a positive control for PPARγ.

### Immunocytochemical analysis

Immunofluorescence experiments were performed as previously described in our laboratory [30] with a few modifications. In brief, cell monolayers grown on glass coverslips were fixed with 4% paraformaldehyde (PFA) for 20 min at room temperature. After fixation, cells were washed in PBS and permeabilized with 0.1% Triton X-100 for 5 min at room temperature. Fixed cells were blocked with 0.1% (m/v) bovine serum albumin and 0.1% (m/v) skim milk in PBS for 1 h before primary antibody incubation for 1.5 h at room temperature or overnight at 4 °C. The rabbit polyclonal (anti-GFAP, 1:200 dilution) and mouse monoclonal (anti-CD11b/c, 1:20) antibodies were used for markers of astrocytes and microglia, respectively. After primary antibody incubation, cells were washed with PBS by gentle agitation and followed by incubation with anti-mouse Alexa Fluor 594 or anti-rabbit Alexa Fluor 488 conjugated secondary antibody (both in 1:500 dilution). Staining in the absence of primary antibodies was used as a negative control. After secondary antibody incubation, cells were washed again with PBS and mounted on a 76 × 26 mm microscope slide (VWR, West Chester, PA) using VECTASHIELD mounting solution containing DAPI. Cells were visualized using a confocal microscope (LSM 700, Carl Zeiss) operated with ZEN software.

### HIV-1_ADA_ gp120 cell treatment

All treatments were performed on monolayers of primary cultures of either mixed rat astrocytes and microglia or pure astrocytes grown in 25-cm^2^ tissue culture flasks. At the beginning of each experiment, culture medium was aspirated and fresh medium containing 5% fetal bovine serum and 5% horse serum with 5 nM HIV_ADA_ gp120 was added. In our pilot experiments, using less than 5 nM of gp120 produced more variable inflammatory responses and concentrations higher than 5 nM did not induce higher responses. Furthermore, in our present study as well as other published reports from our laboratory [6, 7], we have performed MTT assays with different concentrations of gp120 and at 5 nM we did not observe cell toxicity. All experiments were conducted at 37 °C in 5% CO_2_/95% air. Rosiglitazone, pioglitazone and GW9662 were dissolved in DMSO, and a total volume of 5 μL drug solution was added to 5 mL media in T-25 flasks. This volume of DMSO results in a final concentration of 0.001% DMSO. In order to keep conditions consistent between all treatments, we used the same concentration of DMSO in all flasks (e.g., control, vehicle gp120). Cells were treated with PPARγ agonists (1 μM) rosiglitazone or (20 μM) pioglitazone in conjunction with 5 nM gp120. In order to demonstrate specificity of the PPARγ agonists, cells were co-treated with PPARγ specific antagonist GW9662 (500nM). For PPARγ agonists, the doses were selected based on the EC50 values, rosiglitazone, 30–100 nM, and pioglitazone, 500–600 nM (Cayman Chemicals, Ann Arbor, Michigan, USA). Cell suspensions were collected 3 or 6 h after gp120 exposure and prepared for qPCR or immunoblotting analysis as described below.

### Cell viability assay

Cell viability was assessed in primary cultures of rodent glial cells treated with HIV-1_ADA_ gp120 using a standard MTT assay previously described by our laboratory [6]. In brief, cells were plated in 96-well assay plate at a density of 10^5^/well. After 6 h, the medium was aspirated and replaced with fresh medium containing appropriate concentrations of DMSO, gp120, rosiglitazone, or pioglitazone (Additional file 1: Figure S1). These cultures were then incubated for 6 h; the medium was aspirated and replaced with fresh medium containing 10% of MTT (5.0 ng/mL). After 2h incubation, MTT solution was aspirated and 100 μL of DMSO was added to each well. The formazan content of each well was determined by UV spectrophotometry (570 nM) using a SpectraMax 384 microplate reader (Molecular Devices, Sunnyvale, CA).

### Real-time quantitative polymerase chain reaction (qPCR)

Real-time quantitative polymerase chain reaction (qPCR) was applied to determine the transcript levels of inflammatory and oxidative stress markers, PPARγ and GLT-1 according to previously published protocols by our laboratory [8]. Briefly, total RNA was extracted from cell culture or brain regions using TRizol reagent. The concentration of RNA was quantified spectrophotometrically by measuring absorbance at 260 nm. Extracted RNA (2000 ng) was treated with amplification grade DNase I to remove contaminating genomic DNA. The high capacity cDNA reverse transcriptase kit was used to synthesize first-strand cDNA. Rat primers were purchased from ThermoFisher Scientific (Waltham, MA, USA) for the following genes using TaqMan technology: Il-1β (Rn00580432_m1), TNFα (Rn99999017_m1), iNOS (Rn99999069_m1), PPARγ (Rn00440945_m1), GLT-1 (Rn01486045_m1), and cyclophilin B (housekeeping gene; Rn 0835638_m1). Expression levels were normalized to housekeeping gene, cyclophilin B, and compared to saline-treated control group using the comparative Ct (ΔΔCt) method.

### Immunoblot analysis

Western blot analysis was applied according to our previously published protocols to determine the protein expression of PPARγ, phosphorylated forms of NF-κB and GLT-1 [8]. In brief, cell culture and brain tissue homogenates were prepared using a modified RIPA lysis buffer (1% (*v*/*v*) NP-40 in 50 mM tris pH 7.5, 150 mM NaCl, 1 mM ethylene glycol tetraacetic acid (EGTA), 1 mM sodium o-vanadate, 0.25% (*v*/*v*) sodium deoxycholic acid (Doc), 0.1% (*v*/*v*) sodium dodecyl sulfate (SDS), 200 μM phenylmethanesulfonyl fluoride, and 0.1% (*v*/*v*) protease inhibitor cocktail). Samples were sonicated for 10 s and centrifuged at 14,000 rpm for 15 min at 4 °C to remove cellular debris. Nuclear extracts from rat hippocampus were prepared using a nuclear extraction kit from Abcam Inc. (Boston, MA, USA). The extracts were prepared as per the manufacturer’s protocol. Total protein (50 μg) was separated on 10% sodium dodecyl sulfate polyacrylamide gel electrophoresis (SDS-PAGE) and transferred onto a polyvinylidene difluoride membrane. After blocking with 5% skim milk for 2 h, the membrane was probed for protein of interest with primary antibody (rabbit polyclonal anti-PPARγ, 1:1000; rabbit polyclonal anti-phospho-p65 NFκB, 1:100; or rabbit polyclonal anti-EAAT2 which recognizes residues 550 to C-terminus of rat glutamate transporter, 1:1000), and β-actin was used as loading control (mouse monoclonal C4 anti-actin, 1:5000). HRP-conjugated secondary antibody was added after washes in tris-buffered saline with Tween. After further washing, bands were detected using enhanced chemiluminescent reagent. Densitometric analysis was performed in AlphaDigiDoc RT2 software (Alpha Innotech, San Leandro, CA, USA) to quantify relative protein expression. The graphs represent relative density of the bands of interest normalized to corresponding β-actin and calculated fold changes based on control treated group.

### Animals

Adult Wistar male rats, 250–300 g, were purchased from Charles River Laboratories (St. Constant, Quebec, Canada) and were housed at the University of Toronto Division of Comparative Medicine with rodent chow and water on a 12-h light-dark cycle. All procedures were carried out in accordance with the approval of the University of Toronto Animal Care Committee. The rats were randomly assigned to four different groups: saline, gp120, rosiglitazone + gp120, and rosiglitazone + GW9662 + gp120, each group *n* = 6–12 animals.

### Animal surgery and intracerebralventricular (ICV) administration of HIV-1_ADA_ gp120

Sterile stereotaxic technique was performed for all rat brain injections as previously described by our group [8]. In brief, 2–5% isoflurane was used to induce surgical anesthesia. Prior to ICV, animals were administered subcutaneously ketoprofen (5 mg/kg) to induce analgesic effect. HIV-1_ADA_ gp120-associated brain inflammation anesthetized rats were administered a single bilateral ICV injection of HIV_ADA_ gp120 (4 μg/4 μL/ventricle at a rate of 1 μL/min and sacrificed 6, 24, and 72 h post injection. A 5-μL Hamilton syringe was used to inject bilaterally into both ventricles at the following coordinates according to the Atlas of Paxinos and Watson (1986) 0.5 mm posterior to bregma, 1.5 mm lateral from midline, and 3.5 mm ventral from the surface of the skull. Control animals received an equal volume of saline. We have previously demonstrated the specificity of the gp120-mediated inflammatory response through the use of additional controls, (heat-inactivated gp120 and the CCR5 chemokine antagonist maraviroc) [8]. At the designated time points (6, 24, and 72 h) following ICV injection, animals were anesthetized and perfused through the left ventricle of the heart with a 240 mL phosphate buffered saline (PBS). At these time points, brain regions (hippocampus, frontal cortex, and striatum) were collected and harvested for further molecular and biochemical analysis. Samples were flash frozen in liquid nitrogen and kept at −80 °C.

### Intraperitoneal administration of rosiglitazone and GW9662

Animals (*n* = 6–12 per group) were administered through intraperitoneal route (IP), 30 min prior to HIV-1_ADA_ gp120 ICV with rosiglitazone (PPARγ agonist; 10 mg/kg) or co-administration of rosiglitazone with GW9662 (PPARγ antagonist; 5 mg/kg). Both compounds were dissolved in DMSO/saline 1:10. Saline (control) and gp120 (vehicle) animals received the same volume of DMSO/saline 1:10 IP. These compounds are known to effectively permeate across the BBB [31]. The dose selected for rosiglitazone (10 mg/kg) was chosen based on previous reports which demonstrated the neuroprotective effects of rosiglitazone in vivo [23, 25, 26, 31].

### Data analysis

Student’s *t* test was used to determine statistical significance between two groups. Multiple comparisons were performed using one-way ANOVA with Bonferroni’s post-hoc analysis. A *p* value less than 0.05 was considered statistically significant. Data was analyzed using GraphPad Prism software (San Diego, CA). Each set of in vitro experiments were repeated at least three times in cells pertaining to different isolations, and for the in vivo experiments, samples were collected from 4 to 12 animals per group.

## Results

### PPARγ agonists rosiglitazone and pioglitazone reverse HIV-1_ADA_ gp120-mediated inflammatory responses in vitro, in primary cultures of rat mixed astrocytes and microglia

**Fig. 2:**
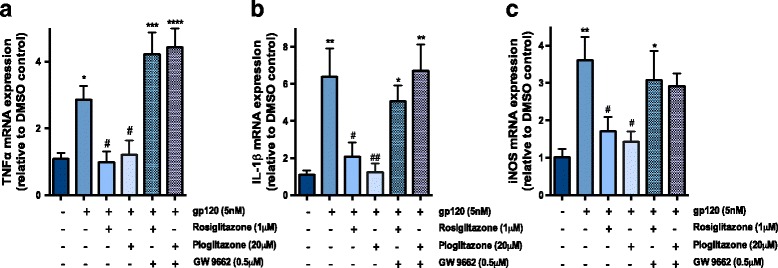
PPARγ agonists rosiglitazone and pioglitazone reverse HIV-1_ADA_ gp120-mediated inflammatory responses in vitro. Primary cultures of mixed rat astrocytes and microglia were treated with PPARγ agonists, rosiglitazone (1 μM) or pioglitazone (20 μM) or co-treated ﻿with ﻿PPARγ antagonist GW9662 (500nM)﻿ for 1 h prior to gp120 (5 nM) exposure for 3 h, and **a** TNF-α, **b** IL-1β, and **c** iNOS mRNA levels were measured using qPCR. Cyclophilin B was used as the housekeeping gene. Results are expressed as mean ± SEM relative to the DMSO (control) of at least 3 separate experiments. Asterisks and pound symbol represent data points significantly different from DMSO (control) and gp120 (vehicle) respectively (*p < 0.05, **p < 0.01, ***p < 0.001, ****p < 0.0001*,* #*p* < 0.05, ##*p* < 0.01) (**a**–**c**)

Previous studies from our laboratory have shown that exposure to HIV-1_96ZM651_ gp120 induces mRNA expression of pro-inflammatory cytokines and oxidative stress markers in primary cultures of astrocytes [6]. Herein, we confirm this inflammatory response using an additional strain of gp120 (ADA) in primary cultures of mixed astrocytes and microglia. These cultures have been characterized through immunocytochemical staining for astrocyte specific marker GFAP and microglia specific marker cd11b/c (Fig. 1). Exposure of the cells to HIV-1_ADA_ gp120 (5 nM) significantly increased the inflammatory markers (TNFα and IL-1β) and indicator of oxidative stress responses (iNOS) at 3 h post gp120 exposure. Treatment with rosiglitazone (1 μM) or pioglitazone (20 μM) significantly reversed the inflammatory responses (Fig. 2). Other doses for rosiglitazone (250 and 500 nM) were tested; however, the 1-μM dose appeared to be the most effective (Additional file 2: Figure S2). For pioglitazone, doses of 1 and 50 μM were also examined, and the 1μM dose appeared too low to exhibit a significant anti-inflammatory effect (Additional file 2: Figure S2). To confirm that the anti-inflammatory effects of PPARγ agonists rosiglitazone and pioglitazone were PPARγ dependent, cells were co-administered with the PPARγ specific antagonist GW9662. As expected, we observed that GW9662 (500 nM) abolished the effects of both agonists (Fig. 2). We performed an MTT assay in primary cultures of mixed astrocytes and microglia to verify that the treatments did not significantly alter cell proliferation and viability; in all cases, cell viability was not significantly different from control (i.e., untreated) cultures (Additional file 1: Figure S1).

### PPARγ agonist rosiglitazone reverses gp120-mediated inflammatory responses in vivo, in an HIV-1_ADA_ gp120 ICV-administered rodent model

**Fig. 3:**
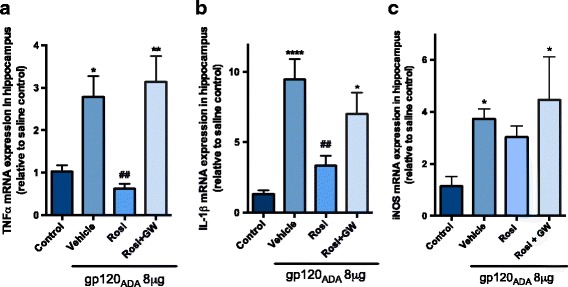
PPARγ agonist rosiglitazone reverses HIV-1_ADA_ gp120-mediated inflammatory responses in hippocampus. Adult Wistar rats were administered IP, 30 min prior to ICV bilateral injection of 4 μg/ventricle HIV-1_ADA_ gp120 with rosiglitazone (10 mg/kg) or co-administration of rosiglitazone with GW9662 (5 mg/kg). Saline (control) and gp120 (vehicle) animals received the same volume of DMSO/saline 1:10 IP. Hippocampus brain regions were isolated 24 h post ICV, and **a** TNFα, **b** IL-1β, and **c** iNOS mRNA levels were measured using qPCR. Cyclophilin B was used as the housekeeping gene. Results are expressed as mean ± SEM relative to saline group (control) *n* = 7–12 animals/group. Asterisks and pound symbol represent data points significantly different from saline (control) and gp120 (vehicle) respectively (*p < 0.05, **p < 0.01, ****p < 0.0001, ##*p* < 0.01)

In our present study, the dose of gp120 for ICV administration (4 μg/ventricle) was chosen based on previous reports using a similar range of doses (1–4 μg/ventricle) injected into the rodent brain to induce an inflammatory effect [32]. In our hands, a single dose of HIV-1_ADA_ gp120 (4 μg/ventricle) induced a significant increase in inflammatory and indicator of oxidative stress responses (TNFα, IL-1β, and iNOS) at 24 h in the hippocampus (Fig. 3) and frontal cortex (Additional file 3: Figure S3).

To evaluate whether PPARγ agonists protect against gp120-induced expression of inflammatory genes TNFα, IL-1β, and indicator of oxidative stress response iNOS, animals administered with gp120 (4 μg/ventricle) were treated with or without an IP dose of 10 mg/kg rosiglitazone. Treatment with PPARγ specific agonist rosiglitazone attenuated gp120-induced expression of TNFα and IL-1β. Although a trend towards reduced expression of iNOS was evident with rosiglitazone treatment, this result did not reach statistical significance (Fig. 3). In order to investigate the specificity of PPARγ mediating the protective effects of rosiglitazone, animals were co-administered with an IP dose of 5 mg/kg PPARγ specific antagonist GW9662. The administration of the antagonist abolished the effects of rosiglitazone in reducing levels of TNFα, IL-1β, and iNOS (Fig. 3). Similar effects were observed in the frontal cortex brain region (Additional file 3: Figure S3).

### PPARγ agonists reverse gp120-mediated downregulation of GLT-1 in vitro, in primary cultures of rat astrocytes, and in vivo, in an HIV-1_ADA_ gp120 ICV-administered rodent model

**Fig. 4:**
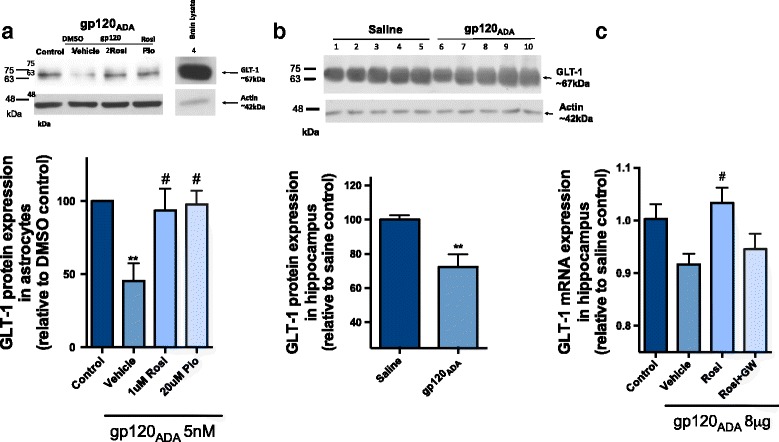
PPARγ agonists reverse HIV-1_ADA_ gp120-mediated downregulation of GLT-1 in vitro and in vivo. Primary cultures of rat astrocytes were treated with PPARγ ligands 1 h prior to gp120 (5 nM) exposure for 6 h, and protein expression of GLT-1 was analyzed through immunoblotting (**a**). Adult Wistar rats were administered IP, 30 min prior to ICV bilateral injection of 4 μg/ventricle HIV-1_ADA_ gp120 with rosiglitazone (10 mg/kg) or co-administration of rosiglitazone with GW9662 (5 mg/kg). Saline (control) and gp120 (vehicle) animals received the same volume of DMSO/saline 1:10 IP. Hippocampus brain regions were isolated 24 h post ICV; protein expression of GLT-1 was analyzed through immunoblotting (**b**), and GLT-1 mRNA levels were measured using qPCR (**c**). For immunoblotting, cell or tissue protein lysate (50 μg) was resolved on a 10% SDS-polyacrylamide gel, transferred to a PVDF membrane. GLT-1 was detected using a rabbit polyclonal antibody (1:1000, dilution). Actin was detected using a mouse monoclonal antibody (1:5000, dilution). Data generated from densitometric analysis is presented as a ratio of GLT-1 expression normalized to actin (loading control). Cyclophilin B was used as the housekeeping gene for qPCR. Results are expressed as mean ± SEM relative to DMSO (control, in vitro) or saline (control, in vivo) of at least 3 separate experiments in vitro and *n* = 5–7 animals/group in vivo*.* Asterisks and pound symbol represent data points significantly different from DMSO or saline (control) and gp120 (vehicle) respectively (**p < 0.01, *p < 0.05, #*p* < 0.05)

Several studies have demonstrated that the glutamate transporter EAAT2 (human) or GLT-1 (rodent) is downregulated in the context of neurological disorders [18, 33]. In particular, gp120 exposure has been reported to decrease functional expression of excitatory amino acid transporter 2 (EAAT2) in primary cultures of human astrocytes [17]. Therefore, in our in vitro and in vivo systems of HIV-1_ADA_ gp120-associated brain inflammation, we investigated levels of the rodent homolog, GLT-1 (Fig. 4). In primary cultures of rat astrocytes, gp120 exposure for 6 h resulted in a significant downregulation of GLT-1 at the protein level, and treatment with PPARγ agonists rosiglitazone (1 μM) or pioglitazone (20 μM) significantly restores the levels (Fig. 4a). This was also observed, in vivo, in the HIV-1_ADA_ gp120 ICV-administered rodent model where we found a significant downregulation of GLT-1 at the protein level 24 h post ICV (Fig. 4b). We were able to further demonstrate that treatment with rosiglitazone restored mRNA levels of GLT-1 in the hippocampus 24 h post ICV (Fig. 4c). Animals were co-administered PPARγ antagonist GW9662 (5 mg/kg) in order to investigate the specificity of PPARγ mediating the protective effects of rosiglitazone. GW9662 administration abolished the effects of rosiglitazone, demonstrating that the restored levels of GLT-1 with rosiglitazone treatment were likely mediated through PPARγ (Fig. 4c).

### PPARγ is downregulated in response to gp120 in vitro, in primary cultures of rat astrocytes, and in vivo, in an HIV-1_ADA_ gp120 ICV-administered rodent model

**Fig. 5:**
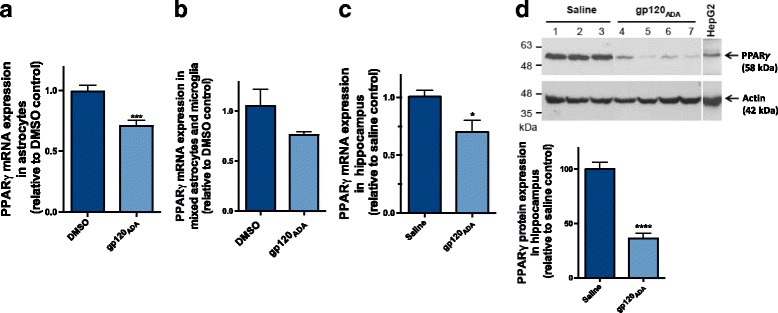
Effect of HIV-1_ADA_ gp120 on the protein and mRNA expression of PPARγ in vitro and in vivo. Primary cultures of rat astrocytes were exposed to g120 (5 nM) for 6 h, and PPARγ mRNA levels were measured using qPCR (**a**). Primary cultures of mixed rat astrocytes and microglia were exposed to g120 (5 nM) for 3 h, and PPARγ mRNA levels were measured using qPCR (**b**). Adult Wistar rats were administered, bilateral ICV, 4 μg/ventricle of gp120; hippocampus was isolated 6–72 h post ICV, and PPARγ mRNA levels were measured using qPCR (**c**), and protein expression of PPARγ was analyzed through immunoblotting (**d**). Cyclophillin was used as the housekeeping gene for qPCR. For immunoblotting, hippocampus tissue protein lysates (50 μg) were resolved on a 10% SDS-polyacrylamide gel and transferred to a PVDF membrane. HepG2 (50 μg) were used as positive control for PPARγ protein. PPARγ was detected using a rabbit polyclonal PPARγ antibody (1:1000 dilution). pt?>Actin was detected using a mouse monoclonal antibody (1:5000, dilution). Data generated from densitometric analysis is presented as a ratio of PPARγ expression normalized to actin (loading control). Results are expressed as mean ± SEM relative to DMSO (control, in vitro) or saline (control, in vivo) of at least 3 separate experiments in vitro and *n* = 6–11 animals/group in vivo*.* Asterisks represent data point significantly different from DMSO or saline (control) (****p < 0.0001, *p < 0.05)

It has been reported that expression of PPARγ can be altered in response to HIV-1 or other inflammatory stimuli [34, 35]. Therefore, we also investigated the expression of PPARγ. In vitro, in primary cultures of rat astrocytes, we observed a significant downregulation of PPARγ at the mRNA level 6 h post gp120 exposure (Fig. 5a) with a similar trend evident in the primary cultures of mixed glial cells exposed to gp120 for 3 h (Fig. 5b). In vivo, mRNA levels of PPARγ were examined at 6 h post ICV, and a significant decrease was also observed in the hippocampus (Fig. 5c). At a later time point, we also investigated the corresponding protein levels and observed a profound downregulation 72 h post ICV (Fig. 5d). Similar data were obtained in the frontal cortex (Additional file 4: Figure S4).

### Involvement of NF-κB redox regulated transcription factor

**Fig. 6:**
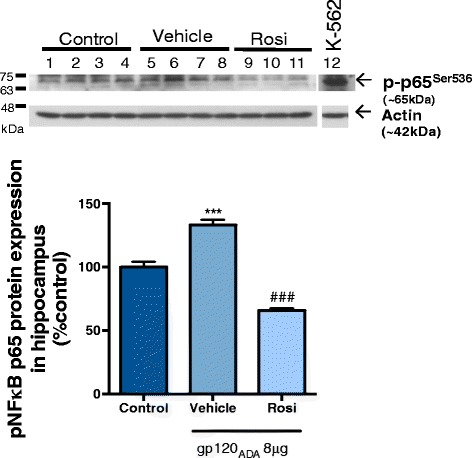
PPARγ agonists reverse HIV-1_ADA_ gp120-induced NF-κB (p-p65) phosphorylation in vivo. Adult Wistar rats were administered IP, 30 min prior to ICV bilateral injection of 4 μg/ventricle HIV-1_ADA_ gp120 with rosiglitazone (10 mg/kg). Saline (control) and gp120 (vehicle) animals received the same volume of DMSO/saline 1:10 IP. Hippocampus brain regions were isolated 5 h post ICV. For immunoblotting, hippocampus tissue nuclear extracts (50 μg) were resolved on a 10% SDS-polyacrylamide gel and transferred to a PVDF membrane. K-562 (immortalized myelogenous leukemia cell line) was used as a positive control for p-p65 protein. NF-κB (p-p65) was detected using a rabbit polyclonal p-p65^Ser536^ (1:100 dilution). Actin was detected using a mouse monoclonal antibody (1:5000, dilution). Data generated from densitometric analysis is presented as a ratio of p-p65^Ser536^ expression normalized to actin (loading control). Results are expressed as mean ± SEM relative to saline group (control) *n* = 3–4 animals/group. Asterisks and pound symbol represent data points significantly different from saline (control) and gp120 (vehicle) respectively (***p < 0.001, *###p* < 0.001)

In order to investigate the involvement of the transcriptional factor NF-κB, animals were administered HIV-1_ADA_ gp120 ICV and rosiglitazone IP but sacrificed at an earlier time point post ICV injection (5 h). The hippocampus tissue was extracted for nuclear proteins, and the phosphorylated levels of the p-65 subunit of NF-κB were analyzed by Western blot. The phosphorylated forms of p-65 correspond to its activation in the nucleus. Our data showed that treatment with rosiglitazone decreased the gp120-induced activation of p-65 in the hippocampus (Fig. 6).

## Discussion

The limitations of currently used ARVs include poor brain penetration, lack of direct anti-inflammatory properties, and neurotoxicity associated with better permeable drugs. Identifying therapeutic compounds that can effectively permeate the BBB and exhibit anti-inflammatory properties may provide an option in treating or preventing HAND.

In this study, we implemented an in vitro model of gp120-associated inflammation using primary cultures of mixed rat astrocytes and microglia exposed to 5 nM R5-tropic HIV-1_ADA_ gp120. R5-tropic strains are considered to be the most prevalent in the brain as the CCR5 co-receptor is expressed on a broad spectrum of cells in the CNS such as microglia, astrocytes, and neurons [36]. The concentration of gp120 in the brain of HIV-1 patients is not clearly documented for obvious reasons of tissue limitation; however, in the periphery (i.e., serum), concentrations of gp120 have been reported to be as high as 92 ng/mL [37]. Immunohistochemical analysis has also detected gp120 in brain tissue from HIV-1-infected patients [38]. Furthermore, studies by Banks and Kastin showed that gp120 crosses the mouse blood brain barrier (BBB) after I.V. administration and the transport is likely mediated through lectin-like mechanisms resembling adsorptive endocytosis. Two hours post I.V. injection of gp120, the percent uptake was 0.15% per gram of brain [39]. Our group has previously demonstrated gp120-mediated inflammatory response in vitro*,* in primary cultures of rodent and human astrocytes, as well as, in vivo in a rodent model of ICV administered HIV-1_ADA_ gp120 [6–8, 40]. Herein, we sought to identify molecular pathways that may play a role in decreasing the gp120-induced acute inflammatory response. PPARγ agonists have been widely used for the treatment of type II diabetes; however, they are also known to exhibit anti-inflammatory and anti-oxidant properties in several models of CNS disorders [24]. It is important to note that although previous reports from randomized clinical trials of rosiglitazone suggested an elevated risk of cardiovascular toxicity, data from the 2009 RECORD trial, a six-year open label randomized control trial with 4447 patients, failed to show risk of overall cardiovascular mortality and morbidity in comparison with other standard type II diabetes medications (metformin, sulfonyl-urea) [41]. In light of these findings, the FDA has removed restrictions from rosiglitazone. Furthermore, we are also using pioglitazone, another PPARγ ligand, which has shown to have reduced cardiovascular risks [42]. To date, a few studies have reported anti-inflammatory potential of PPARγ agonists in attenuating HIV-associated inflammatory response [5, 26, 43]. Although it has been reported that rosiglitazone treatment in primary cultures of rodent astrocytes attenuated LPS-induced secretion of several pro-inflammatory markers (IL-12, TNFα, IL-1β, IL-6, MCP-1 [44, 45], this effect has not been thoroughly examined in astrocytes or microglia in the context of gp120-associated pathologies. Our in vitro data demonstrated that glial treatment with either rosiglitazone or pioglitazone reversed the gp120-mediated inflammatory responses. Furthermore, we showed that co-administration of a synthetic PPARγ antagonist, GW9662, which acts as a potent, irreversible, and selective PPARγ antagonist by modifying a cysteine residue in the ligand-binding site of PPARγ, abolished the rosiglitazone or pioglitazone anti-inflammatory effects, suggesting that these effects are specifically mediated by PPARγ.

Next, we sought to characterize an acute in vivo model of HIV-1-associated brain inflammation by ICV administration of HIV-1_ADA_ gp120. A single bilateral ICV dose of (4 μg/ventricle) resulted in a significant induction of inflammatory genes (TNFα, IL-1β, iNOS) at 24 h in the hippocampus (Fig. 3) and frontal cortex (Additional file 3: Figure S3). Our group has previously demonstrated that this inflammatory response is mediated through direct interaction of R5-tropic gp120 with chemokine co-receptor CCR5 in vitro and in vivo [6, 8].

We then investigated the anti-inflammatory properties of rosiglitazone in our in vivo rodent model of HIV-1_ADA_ gp120-induced brain inflammation. Our results showed that treatment with 10 mg/kg rosiglitazone reduced gp120-induced gene expression of inflammatory markers (TNFα and IL-1β) in hippocampus (Fig. 3). These results are in agreement with other studies that have observed rosiglitazone-mediated downregulation of inflammatory cytokines in various in vivo models of CNS disorders [23]. To the best of our knowledge, only two studies have examined the in vivo therapeutic efficacy of PPARγ agonists in the context of HIV-1 infection in the brain. Huang et al. showed that rosiglitazone treatment reduced HIV-1 viral protein Tat increase in BBB permeability, astrogliosis, and neuronal loss [5]. Potula et al. used a severe combined immunodeficiency mouse model of HIV-1 encephalitis, where treatment of rosiglitazone resulted in suppression of viral replication in brain macrophages [25]. The anti-inflammatory effects of rosiglitazone in our gp120 model were abolished with co-administration of 5 mg/kg GW9662 (PPARγ antagonist), demonstrating that the anti-inflammatory effects are specifically mediated by PPARγ (Fig. 3).

Glutamate is the most abundant neurotransmitter in mammalian CNS. Clearance of glutamate from the extracellular space is regulated by specific uptake transporters existing in the plasma membrane of glial cells and neurons. Many cell types throughout the brain express glutamate transporters; however, uptake by astrocytes is quantitatively the most significant [46]. EAAT2/GLT-1 is ﻿the primary transporter ﻿ responsible for glutamate uptake in the mammalian brain, and impairment to this transporter can result in glutamate excitotoxicity which has been proposed to contribute to several neurological diseases including HAND [47]. It has been reported that HIV-1-infected individuals have five-fold greater levels of glutamate in the CSF compared to healthy controls [48]. More recently, studies investigating glutamate levels in patients receiving combinational antiretroviral therapy observed selective increases in glutamate CSF levels in patients with HAND compared to those without neurological impairments [16]. It has been proposed that gp120 may play an important role in regulating the glutamate transporter by decreasing its functional expression in primary cultures of human astrocytes [17] and in gp120 transgenic mice [18]. In addition, others have reported that gp120 may be disrupting ion fluxes across the plasma membrane [49] or stimulating glutamate release [19]. Our data corroborate those of other groups who have demonstrated decreased expression of GLT-1 after gp120 exposure [17, 18]. This effect has been proposed to be mediated by TNFα which could transcriptionally repress GLT-1 through activation of the NF-κB pathway [50].

Strategies to modulate glutamate excitotoxicity include the use of memantine, an *N*-methyl-d-aspartate (NMDA) receptor antagonist; however, initial clinical trials in patients with HAND were unsuccessful [51]. Other on-going strategies include regulation of enzymes that are responsible for producing glutamate [52] or transporters involved with glutamate release [53]; however, currently, no clinically available brain-penetrating compounds exist. To date, a limited number of studies have investigated PPARγ as a target for GLT-1 regulation [33, 54]. In light of these recent reports demonstrating that PPARγ activation increases astrocytic GLT-1 expression in the context of ischemia and glioma cells, we showed that targeting PPARγ for GLT-1 modulation is also applicable in the context of HIV-1-associated brain inflammation. The mechanism for restored GLT-1 expression could be due to the anti-inflammatory effects of these agonists which we have shown to attenuate TNFα release and activation of the NF-κB pathway (Fig. 6). In addition, previous bioinformatic analyses revealed that there are at least 6 putative consensus PPAR response element sites in the promoter region of the EAAT2 gene and in vitro treatment with rosiglitazone increased promoter activity [33].

We also examined PPARγ expression following gp120 administration. A profound downegulation of PPARγ was observed at both the mRNA and protein levels after gp120 exposure (Fig. 5). Similar downregulatory effects on PPARγ has previously been shown in other tissues and cell systems of inflammatory diseases [34, 55]. Furthermore, downregulation of this nuclear receptor has been demonstrated in lung tissue in the context of HIV-1-associated interstitial pneumonitis [56] and chronic obstructive pulmonary disease [57]. Recently, immunohistochemical analysis of feline immunodeficiency virus animal brains and HIV-1-infected post-mortem brain tissue revealed a reduced expression of PPARγ [35]. In addition, it has also been reported that PPARγ can be downregulated through activation of the mitogen-activated kinases such as extracellular signal-regulated kinases 1 and 2 (ERK1/2) which can phosphorylate the activation function resulting in negative feedback [58]. We have previously demonstrated that this pathway is activated in response to gp120 [8]. Together, these studies including our own suggest the involvement of this nuclear receptor in HIV-1-mediated inflammatory response.

To investigate the mechanisms which could be involved in the PPAR-mediated anti-inflammatory effects, we examined the effect of rosiglitazone treatment on the suppression of redox regulated transcriptional factor NF-κB upregulated by gp120. NF-κB binding sites have been reported in several promoter regions of inflammatory cytokine genes [59], and two binding sites have also been identified in the promoter-proximal enhancer region of HIV-1 LTR [60]. Several mechanisms have been reported for the PPARγ-mediated inhibition of NF-κB; these mechanisms include physical interaction of PPARγ with NF-κB, co-activator competition of both transcriptional factors regulation of protein localization, and prevention of signal dependent clearance of co-repressor complexes on inflammatory promoters [28, 29]. Our data suggest that treatment with PPARγ agonist rosiglitazone in vivo decreases significantly the gp120-induced phosphorylation of NF-κB in the hippocampus.

## Conclusion

Findings from our in vitro and in vivo work revealed that PPARγ is an important pathway involved in HIV-1 brain-associated inflammation and could constitute a potential molecular target in the treatment/prevention of HIV-1 brain inflammation and HAND.

## Additional files

Additional file 1: Figure S1. Effect of DMSO, HIV-1_ADA_ gp120, PPARγ agonists on cell viability in vitro. Primary cultures of mixed rat astrocytes and microglia were treated with either DMSO, gp120 (5 nM), rosiglitazone (1 μM), pioglitazone (20 μM, 50 μM) or GW9662 (500 nM) for 6 h, and cell viability was assessed using MTT assay. Results are expressed as percent of control and reported as mean ± SEM of at least 3 separate experiments (PDF 25 kb)

Additional file 2: Figure S2. PPARγ agonists rosiglitazone and pioglitazone reverse HIV-1_ADA_ gp120-mediated inflammatory responses in vitro*.* Primary cultures of mixed rat astrocytes and microglia were treated with PPARγ agonists, rosiglitazone (250 nM–1 μM) or pioglitazone (1–50 μM) for 1 h prior to gp120 (5 nM) exposure for 3 h and. (A) TNF-α, (B) IL-1β, and (C) iNOS mRNA levels were measured using qPCR. Cyclophilin B was used as the housekeeping gene. Results are expressed as mean ± SEM relative to DMSO of at least 3 separate experiments. Asterisks and pound symbol represent data points significantly different from DMSO (control) and gp120 (vehicle) respectively (*p < 0.05, **p < 0.01, *#p* < 0.05, *##p* < 0.01) (A-C) (PDF 47 kb)

Additional file 3: Figure S3. PPARγ agonist rosiglitazone reverses HIV-1_ADA_ gp120-mediated inflammatory responses in frontal cortex. Adult Wistar rats were administered IP, 30 min prior to ICV bilateral injection of 4 μg/ventricle HIV-1_ADA_ gp120 with rosiglitazone (10 mg/kg) or co-administration of rosiglitazone with GW9662 (5 mg/kg). Saline (control) and gp120 (vehicle) animals received the same volume of DMSO/saline 1:10 IP. Frontal cortex brain regions were isolated 24 h post ICV and (A) TNFα and (B) IL-1β and indicator of oxidative stress response (C) iNOS mRNA levels were measured using qPCR. Cyclophillin was used as the housekeeping gene. Results are expressed as mean ± SEM relative to saline group (control) *n* = 7–12 animals/group. Asterisks and pound symbol represent data points significantly different from saline (control), and gp120 (vehicle) respectively. (*p < 0.05, **p < 0.01, *#p* < 0.05*, ####p* < 0.0001) (PDF 67 kb)

Additional file 4: Figure S4. Effect of HIV-1_ADA_ gp120 on the mRNA and protein expression of PPARγ in frontal cortex. Adult Wistar rats were administered, bilateral ICV, 4 μg/ventricle of gp120, brain tissue was isolated 6–72 h post ICV. PPARγ mRNA expression was measured using qPCR. Cyclophillin was used as the housekeeping gene. For immunoblotting, frontal cortex tissue protein lysates (50 μg) were resolved on a 10% SDS-polyacrylamide gel and transferred to a PVDF membrane. HepG2 (50 μg) cells were used as positive control for PPARγ protein. PPARγ was detected using a rabbit polyclonal PPARγ antibody (1:1000 dilution). Actin was detected using a mouse monoclonal antibody (1:5000, dilution). Data generated from densitometric analysis is presented as a ratio of PPARγ expression normalized to actin (loading control). Results are expressed as mean ± SEM relative to saline group (control) *n* = 4–12 animals/group*.* Asterisks represent data point significantly different from saline (control) animals (*p < 0.05, **p < 0.01) (PDF 94 kb)

## Abbreviations

ARVs: Antiretroviral drugs
BBB: Blood-brain barrier
CNS: Central nervous system
CSF: Cerebrospinal fluid
DAPI: 4′,6′-diamidino-2-phenylindole hydrochloride
Doc: Deoxycholic acid
EAAT2: Excitatory amino acid transporter 2
EGTA: Ethylene glycol tetraacetic acid
ERK: Extracellular regulated kinase
GLT-1: Glutamate transporter 1
gp120: Glycoprotein 120
GW9662: 2-Chloro-5-nitro-*N*-phenylbenzamide
HAART: Highly active antiretroviral therapy
HAND: HIV-associated neurocognitive disorder
HIV: Human immunodeficiency virus
ICV: Intracerebralventricular
IL-1β: Interleukin-1beta
IL-6: Interleukin-6
IL-8: Interleukin-8
iNOS: Inducible nitric oxide synthase
IP: Intraperitoneal
LPS: Lipopolysaccharide
NF-κB: Nuclear factor kappa B
NO: Nitric oxide
PBS −/−: Phosphate-buffered saline without Ca2+ and Mg+
PPARγ: Peroxisome proliferator-activated receptor gamma
ROS: Reactive oxygen species
SDS: Sodium dodecyl sulfate
Tat: Transactivator of transcription
TNFα: Tumor necrosis factor alpha
Vpr: Viral protein R
