# Supplementary figures and images for: Peroxisome proliferator-activated receptor-gamma: potential molecular therapeutic target for HIV-1-associated brain inflammation
Source: J Neuroinflammation. 2017 Sep 8;14:183. doi: 10.1186/s12974-017-0957-8 (PMC5591559; doi:10.1186/s12974-017-0957-8)

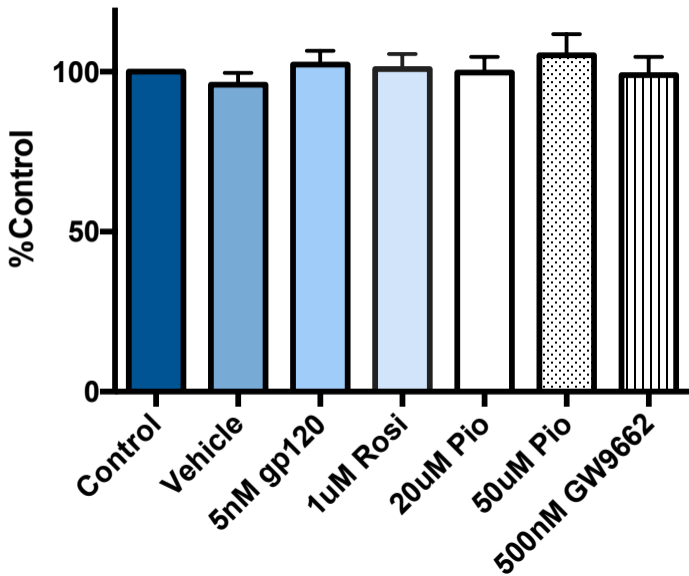

Supplement: Supplementary file 1 — Effect of DMSO, HIV-1ADA gp120, PPARγ agonists on cell viability in vitro. Primary cultures of mixed rat astrocytes and microglia were treated with either DMSO, gp120 (5 nM), rosiglitazone (1 μM), pioglitazone (20 μM, 50 μM) or GW9662 (500 nM) for 6 h, and cell viability was assessed using MTT assay. Results are expressed as percent of control and reported as mean ± SEM of at least 3 separate experiments (PDF 25 kb) [file 12974_2017_957_MOESM1_ESM.pdf]

A

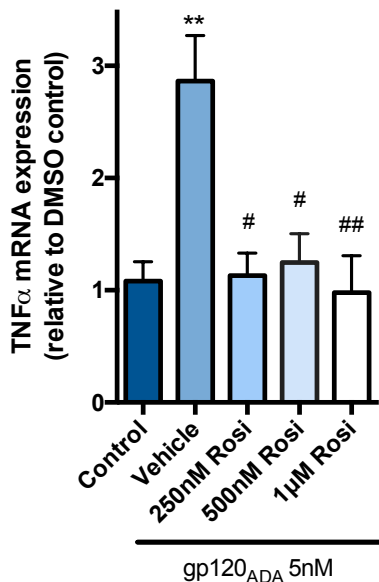

B

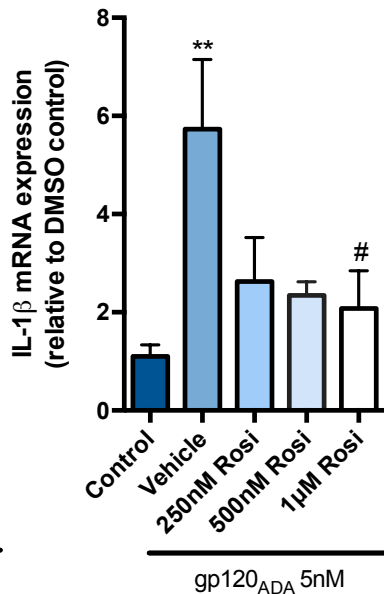

C

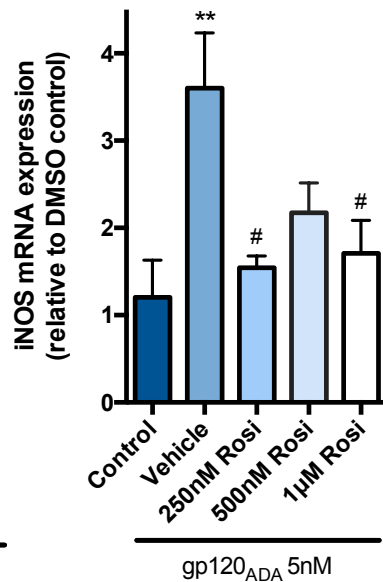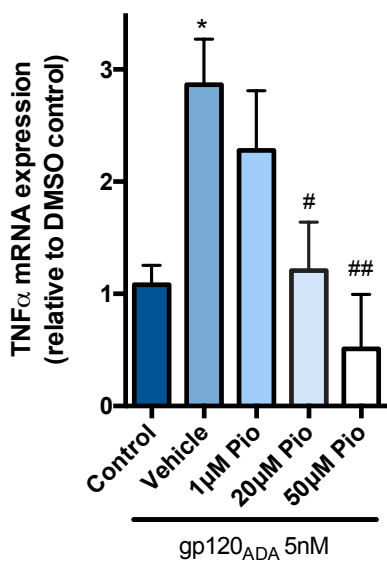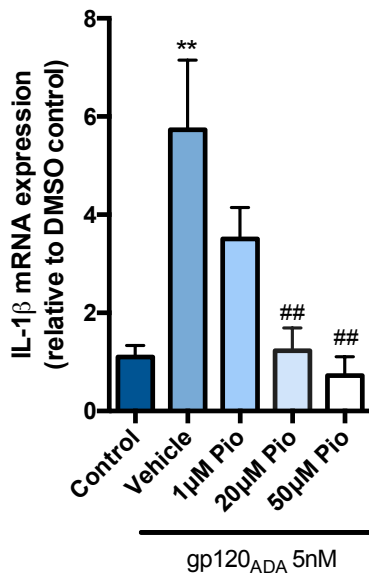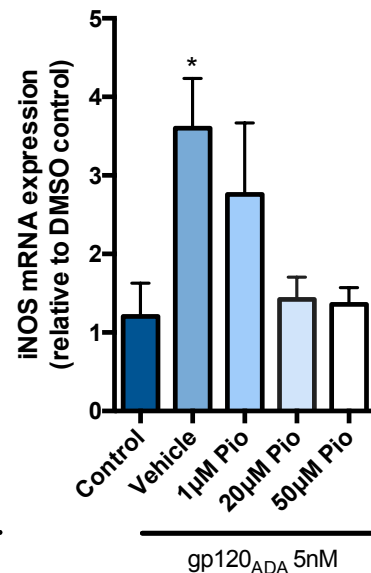

Supplement: Supplementary file 2 — PPARγ agonists rosiglitazone and pioglitazone reverse HIV-1ADA gp120-mediated inflammatory responses in vitro. Primary cultures of mixed rat astrocytes and microglia were treated with PPARγ agonists, rosiglitazone (250 nM–1 μM) or pioglitazone (1–50 μM) for 1 h prior to gp120 (5 nM) exposure for 3 h and. (A) TNF-α, (B) IL-1β, and (C) iNOS mRNA levels were measured using qPCR. Cyclophilin B was used as the housekeeping gene. Results are expressed as mean ± SEM relative to DMSO of at least 3 separate experiments. Asterisks and pound symbol represent data points significantly different from DMSO (control) and gp120 (vehicle) respectively (*p < 0.05, **p < 0.01, #p < 0.05, ##p < 0.01) (A-C) (PDF 47 kb) [file 12974_2017_957_MOESM2_ESM.pdf]

**A**

**PPAR $\gamma$  mRNA expression  
in frontal cortex  
(relative to saline control)**

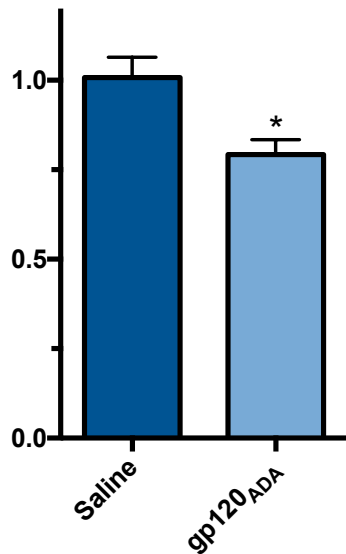**B**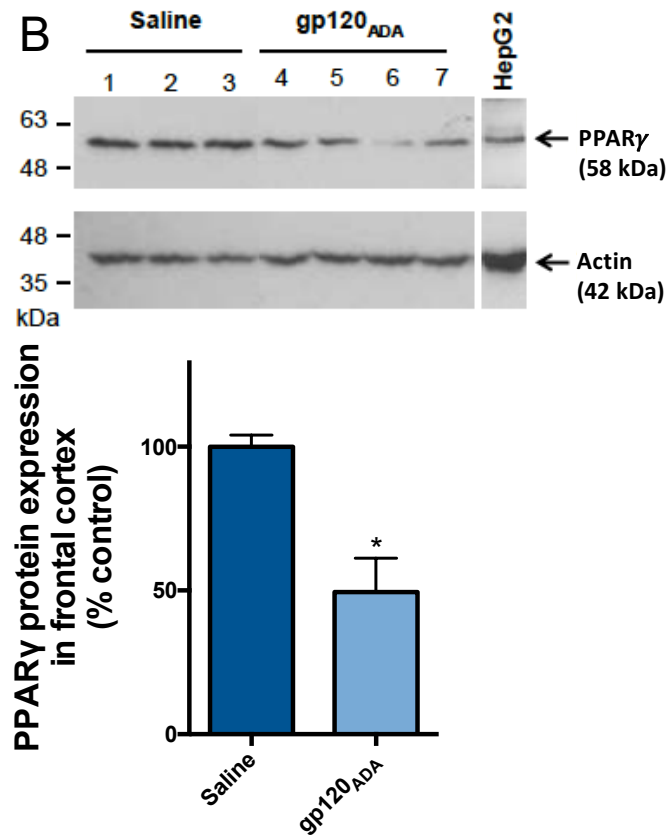

Supplement: Supplementary file 4 — Effect of HIV-1ADA gp120 on the mRNA and protein expression of PPARγ in frontal cortex. Adult Wistar rats were administered, bilateral ICV, 4 μg/ventricle of gp120, brain tissue was isolated 6–72 h post ICV. PPARγ mRNA expression was measured using qPCR. Cyclophillin was used as the housekeeping gene. For immunoblotting, frontal cortex tissue protein lysates (50 μg) were resolved on a 10% SDS-polyacrylamide gel and transferred to a PVDF membrane. HepG2 (50 μg) cells were used as positive control for PPARγ protein. PPARγ was detected using a rabbit polyclonal PPARγ antibody (1:1000 dilution). Actin was detected using a mouse monoclonal antibody (1:5000, dilution). Data generated from densitometric analysis is presented as a ratio of PPARγ expression normalized to actin (loading control). Results are expressed as mean ± SEM relative to saline group (control) n = 4–12 animals/group. Asterisks represent data point significantly different from saline (control) animals (*p < 0.05, **p < 0.01) (PDF 94 kb) [file 12974_2017_957_MOESM4_ESM.pdf]
