# Supplementary material for: Peroxisome proliferator-activated receptor-gamma: potential molecular therapeutic target for HIV-1-associated brain inflammation
Source: J Neuroinflammation. 2017 Sep 8;14:183. doi: 10.1186/s12974-017-0957-8 (PMC5591559; doi:10.1186/s12974-017-0957-8)
Supplement: Supplementary file 3 — PPARγ agonist rosiglitazone reverses HIV-1ADA gp120-mediated inflammatory responses in frontal cortex. Adult Wistar rats were administered IP, 30 min prior to ICV bilateral injection of 4 μg/ventricle HIV-1ADA gp120 with rosiglitazone (10 mg/kg) or co-administration of rosiglitazone with GW9662 (5 mg/kg). Saline (control) and gp120 (vehicle) animals received the same volume of DMSO/saline 1:10 IP. Frontal cortex brain regions were isolated 24 h post ICV and (A) TNFα and (B) IL-1β and indicator of oxidative stress response (C) iNOS mRNA levels were measured using qPCR. Cyclophillin was used as the housekeeping gene. Results are expressed as mean ± SEM relative to saline group (control) n = 7–12 animals/group. Asterisks and pound symbol represent data points significantly different from saline (control), and gp120 (vehicle) respectively. (*p < 0.05, **p < 0.01, #p < 0.05, ####p < 0.0001) (PDF 67 kb) [file 12974_2017_957_MOESM3_ESM.pdf]

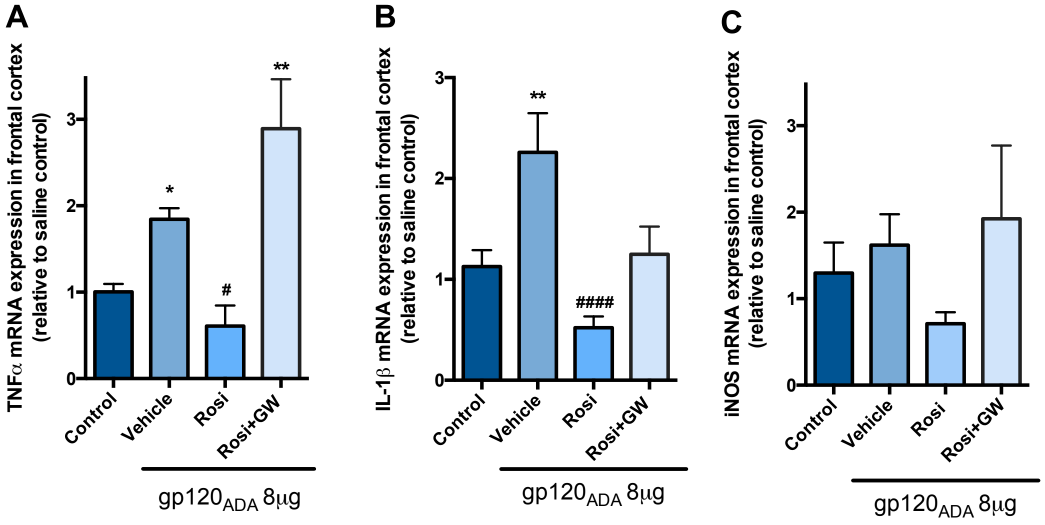

A
TNFα mRNA expression in frontal cortex (relative to saline control)
0
1
2
3
*
#
**
Control
Vehicle
Rosi
Rosi+GW
gp120ADA 8μg
B
IL-1β mRNA expression in frontal cortex (relative to saline control)
0
1
2
3
**
####
Control
Vehicle
Rosi
Rosi+GW
gp120ADA 8μg
C
iNOS mRNA expression in frontal cortex (relative to saline control)
0
1
2
3
Control
Vehicle
Rosi
Rosi+GW
gp120ADA 8μg
